# Toward a Better Classification System for NK-LGL Disorders

**DOI:** 10.3389/fonc.2022.821382

**Published:** 2022-02-01

**Authors:** Gaëlle Drillet, Cédric Pastoret, Aline Moignet, Thierry Lamy, Tony Marchand

**Affiliations:** ^1^ Service d’Hématologie Clinique, Centre Hospitalier Universitaire de Rennes, Rennes, France; ^2^ Laboratoire d’Hématologie, Centre Hospitalier Universitaire de Rennes, Rennes, France; ^3^ Faculté de Médecine, Université Rennes 1, Rennes, France; ^4^ CIC 1414, Centre Hospitalier Universitaire de Rennes, Rennes, France; ^5^ Institut National de la Santé et de la Recherche Médicale (INSERM) U1236, Rennes, France

**Keywords:** chronic lymphoproliferative disorders of NK cells, NK cells, KIR phenotype, STAT3, large granular lymphocyte leukemia

## Abstract

Large granular lymphocytic leukemia is a rare lymphoproliferative disorder characterized by a clonal expansion of T-lineage lymphocyte or natural killer (NK) cells in 85 and 15% of cases respectively. T and NK large granular leukemia share common pathophysiology, clinical and biological presentation. The disease is characterized by cytopenia and a frequent association with autoimmune manifestations. Despite an indolent course allowing a watch and wait attitude in the majority of patients at diagnosis, two third of the patient will eventually need a treatment during the course of the disease. Unlike T lymphocyte, NK cells do not express T cell receptor making the proof of clonality difficult. Indeed, the distinction between clonal and reactive NK-cell expansion observed in several situations such as autoimmune diseases and viral infections is challenging. Advances in our understanding of the pathogenesis with the recent identification of recurrent mutations provide new tools to prove the clonality. In this review, we will discuss the pathophysiology of NK large granular leukemia, the recent advances in the diagnosis and therapeutic strategies.

## Introduction

Large granular lymphocytic (LGL) leukemia is a rare disease that accounts for 2 to 5% of chronic lymphoproliferative disorders (1). Its incidence is probably underestimated in view of its indolent and often asymptomatic course and diagnostic difficulties. LGL leukemia is mainly characterized by cytopenia, primarily neutropenia predisposing to infections and is frequently associated with an array of autoimmune diseases, in particular rheumatoid arthritis. There are two main subtypes of LGL leukemia, respectively with a T or NK phenotype and a respective incidence of 85 and 15%. A provisional entity so-called chronic lymphoproliferative disorders of NK cells, or CLPD-NK, was included in the last WHO classification in 2016 (2) as a means of distinguishing it from EBV induced aggressive NK-LGL leukemia whose prognosis is quite poor.

LGL leukemia needs to be distinguished from reactive LGL proliferation, which is frequent, particularly in the context of viral infections, autoimmune diseases, after splenectomy or in post-transplant patients. Diagnosis of LGL leukemia is based on two mandatory criteria which help to differentiate it from reactive LGL lymphocytosis: cytological identification of lymphocytes with granules > 0.5 G/L observed at least over 6 months and proof of clonality. T-LGL clonality is easily demonstrated by TCR rearrangement. On the other hand, NK-LGL clonality is far more complex to identify, as NK cells do not express CD3 on their surface and lack the T cell antigen receptor (TCR). In this review, we develop advances in the pathophysiology and understanding of NK-LGL leukemia. We review recent progresses in the development of tools for clonality diagnosis that can help to optimize nosological classification of chronic NK proliferations before finally considering therapeutic strategies.

## NK Cell: A Lymphocyte With Cytotoxic Capabilities and With Complex Activation Modalities

NK cells have a cytotoxic and cytokinic activity close to that of the CD8+ cytotoxic T lymphocyte directed against aberrant autologous cells (infected, tumoral ou stressed) giving them antiviral and anti-tumoral functions. In contrast to T cells, NK cells do not express the TCR-CD3 complex on their surface. On the other hand, they do express the CD16a molecule, a low affinity type IIIA immunoglobulin constant fragment receptor, which enables them to bind and opsonized cells. NK cells also express CD56, or neural cell adhesion molecule (NCAM), which is more broadly expressed by other extra-hematopoietic cell types, and by a minority of activated cytotoxic T cells. They are also routinely included in CD2+/CD5-/CD7+ lymphocyte flow cytometry analysis panels (3).

Two types of NK cells with different functions have been historically identified through differential expression of CD56, CD16 in flow cytometry (4).

1) CD56high CD16low NK cells are mainly cytokine-producing NK cells such as interferon gamma. The production of interferon gamma by NK cells is stimulated by IL-12 and IL-18 in synergy with IL-2 and IL-15, which promote NK cell activation more broadly (5).

2) CD56low CD16high NK cells have a mainly cytotoxic function. These lymphocytes are the main agent of antibody-dependent cell-mediated cytotoxicity (ADCC) *via* CD16. After activation of the NK cell, targeted cells apoptosis can be mediated by two NK cell cytotoxicity mechanisms, also used by T cells, namely the perforin-granzyme pathway and the Fas/Fas ligand pathway. The perforin released by exocytosis from NK cells create pores in the plasma membrane of the targeted cell enabling granzymes entry. Granzyme B leads to the activation of the caspase cascade (6) while granzyme A induces cell death by a mitochondrial caspase-independent mechanism (7). The FAS/FASL complex or TRAIL/TRAIL-Rs induce apoptosis by pathways similar to granzyme B.

NK lymphocyte can still be considered as part of innate immunity since it uses a repertoire of surface receptors, is germline-encoded, and able to recognize stressed cells, without the need for prior sensitization and to act immediately. NK lymphocytes recognize not only MHC class I or MHC class I mimicking molecules, but also other molecules. Ligand specificity is to a variable extent dependent on the type of receptor. These receptors can induce activating or inhibitory signals to the NK and are not specific to them since they are also expressed by T lymphocytes (8).

Among the receptors that recognize the classical MHC class I (HLA-A, B, C), are the Killer Immunoglobulin-like Receptors (KIR), each of which recognizes an HLA subtype with a relatively high specificity. Every single NK lymphocyte expresses a few KIR receptors among the existing KIR, coded by 15 genes on chromosome 19, with a high polymorphism, frequent chromosomal recombinations and alternative splicing (9, 10). KIRs can induce either an inhibitory or an activating signal. The lectin-like receptors of the CD94-NKG2 heterodimer are another large NK receptor family. CD94-NKG2A induce an inhibitory signal through the non-classical MHC class I (HLA-E), which has a more restricted polymorphism than the classical MHC type I (11). Natural Cytotoxicity Receptors (NCRs), mainly represented by NKp46, NKp30, and NKp44, recognize non-MHC molecules on the cell-surface or secreted by tumoral or virus-infected cells. They also have an activating role for NK lymphocytes (12).

NK cell activation is more complex than TCR/BCR antigenic activation, which are present on T cells and B cells respectively. NK cell activation is determined by the integration of multiple signals from these different surface receptors and is only possible when the sum of activating signals exceeds that of inhibiting signals. Activation depends on the number of receptors, their affinity and the inhibitory threshold of the cell.

## Pathophysiology of NK-LGL Leukemia

**Figure 1:**
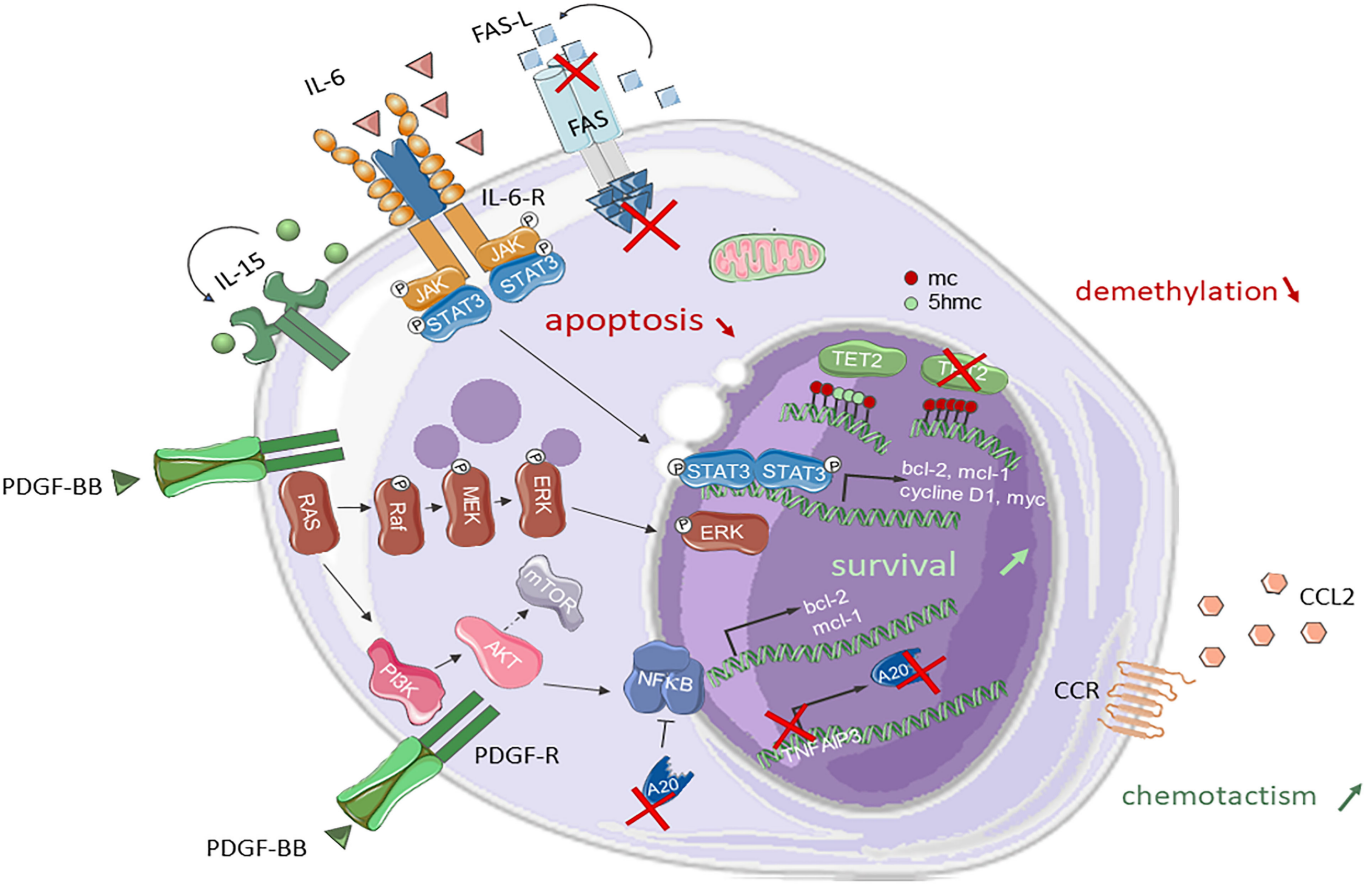
Signaling pathways and mutations involved in NK-LGL leukemia pathogenesis. STAT3, RAS/MAPK and PI3K/AKT pathways are constitutionally activated in NK-LGL leukemia. The PI3K/AKT pathway leads to the activation of m-TOR and NFκB. STAT3 and NFκB promote the transcription of anti-apoptotic genes such as *bcl-2* ou *mcl-1*. TNFAIP3 can undergo an inactivating mutation of the A20 protein that negatively regulates NFκB. LGLs are resistant to Fas mediated apoptosis. A *TET2* loss-of-function mutation is found in 34% of NK-LGLs. The gene encoding the chemokine CCL22 is mutated in 20% of NK-LGLs. Fas, First Apoptosis Signal; FasL, FasLigand; IL, interleukin; IL-R, interleukin-receptor; Jak, Janus Kinase; STAT3, Signal transducer and activator of transcription 3; PDGF-BB, platelet-derived growth factor BB; MEK, mitogen activated protein kinase; ERK, extracellular-signal-regulated kinase; PI3K, phosphatidyl Inositol 3-Kinase; mTOR, mammalian target of rapamycin; NFκB, nuclear factor kappa B; Mcl1, Myeloid cell leukemia1; Bcl, B-cell lymphoma 2; CCL22, C-C Motif Chemokine Ligand 22; 5-mc, 5-methylcytosin; 5hmc, 5-hydroxymethylcytosin; TET2, Ten-eleven-translocation 2.

The cytotoxic function, characteristic of both T and NK cells explains the common pathophysiological basis of T- and NK-LGL leukemia. The development of LGL leukemia is probably secondary to a chronic stimulation induced by a viral infection or a public antigen. Autocrine and paracrine interleukin 15 plays a central role in the proliferation of NK cells (13), which is initially polyclonal and then switches to monoclonal proliferation through selection of an NK clone with an activated KIR profile contrasting with the mainly inhibitory profile of KIRs observed in physiological situations (14, 15). The development of leukemia is also the consequence of dysregulated activation of several anti-apoptotic signaling and cell survival pathways (**Figure 1**). The JAK/STAT pathway plays a central role in the pathophysiology of NK-LGL leukemia. Constitutive activation of STAT3 was initially reported in 2001 (16) and an activating mutation of *STAT3* was identified in the SH2 domain on two predominant hotspots (D661 and Y640) in LGL leukemia (17), as well as in NK/T and ATLL lymphomas (18). This mutation induces constitutive phosphorylation and STAT3 unit dimerization leading to the transcription of anti-apoptotic genes such as Mcl-1 belonging to the Bcl-2 family. The *STAT3* mutation is found in 30% of NK-LGL leukemia as well as in 30-40% of T-LGL leukemia, linking the two entities (17, 19). The introduction of the *in vitro* STAT3 inhibitor AG-490 or STAT3 antisense oligonucleotide treatment shows restoration of apoptosis of clonal NK LGLs apoptosis (16). A significant proportion of unmutated STAT3 LGL leukemia cases also features hyperactivation of the JAK/STAT pathway by two mechanisms (19); i) underexpression of the *SOCS3* (suppressor of cytokine signaling-3) gene, ii) excess autocrine production of interleukin-6 by NK-LGLs. Deleterious mutations of the JAK/STAT pathway were described by whole exome sequencing, such as PTK2/FAK1, PIK3R1 (20, 21), FLT3 and CD40 ligand (22). Constitutive activation of STAT3 and production of IL6 induce an increase in the transcription and expression of Fas ligand in LGL leukemia. However, NK-LGL leukemic cells show resistance to the pro-apoptotic signal of the Fas ligand (23–25) without any gene mutation being identified. Clonal LGL-NKs produce a soluble variant FAS, thought to act as a soluble FAS receptor, blocking the FAS-ligand (26). Moreover, there is a certain correlation between the soluble Fas ligand concentration and the depth of neutropenia in LGL leukemia, suggesting that the soluble Fas ligand plays a role in neutrophil apoptosis (27). The MAP kinase pathway also participates in the dysregulation of the balance between survival and apoptosis in NK-LGL leukemia (28). Suppression of ERK (extracellular signal-regulated kinase) activity by a MEK inhibitor reduces NK-LGL survival. The same phenomenon is observed with the inhibition of Ras, reported to be constitutively activated in NK-LGL leukemia patients. *KRAS, NOTCH1* and *PTEN* mutations were found in different cohorts (20, 21, 29). The PI3K-Akt complex, which can be activated by Ras and inhibited by PTEN, is also deregulated in LGL leukemia (30). Akt has numerous downstream targets involved in the cell cycle, including mTOR. Mutations in *PIK3R1*, *PIK3CD* and PIK3AP1 genes have been also identified for instance (21, 31). Another recurrent mutation affecting TNFAIP3 (tumor necrosis factor alpha–induced protein 3) was identified in 5% of LGL leukemia patients (31). This mutation results in negative regulation of NFκB signaling. The A20 protein encoded by TNFAIP3 inhibits NFB by ubiquitination mechanism. NFκB is constitutively activated in LGL leukemia (32). Downstream of the PI3K-Akt pathway, NFκB causes an increase in the anti-apoptotic factor Mcl-1, independently of STAT3. PDGF-β (Platelet-derived growth factor subunit Beta) produced in excess by clonal LGLs, forms an anti-apoptotic autocrine loop, activating the signaling pathways mentioned above, PI3K-AKT, RAS/MEK1/ERK, and JAK/STAT. Its inhibition by a neutralizing antibody *in vitro* leads to a decrease in AKT phosphorylation (33). We recall the role of IL-15 and its receptor produced in excess in LGL-NK leukemias (34). Transgenic mice overexpressing IL-15 by post-transcriptional regulation defect developed NK lymphocyte proliferation and secondary aggressive LGL-NK leukemia rapidly lethal (35, 36). More recently, mutations in the CCL22 gene have been described in 20% of LGL-NK leukemia, and are specific to the NK subtype and exclusive of other mutations (37). The *CCL2* mutation induces *in vitro* increased CCL2 chemotaxis and decreased internalization of its Th2 T cell receptor CCR4. CCL2-mutated NK LGLs show higher CD56 expression than non-mutated ones (38).

Finally, epigenetic modifications in NK-LGL leukemia were discovered more recently. A TET2 mutation was identified in approximately 30% of patients with NK-LGL leukemia in three successive series (21, 29, 31). In Olson’s 7-patient cohort, 5 times more methylated regions were observed in clonal NK-LGLs than in normal NK cells in reduced-representation bisulfite sequencing data (31), involving over a hundred RNA polymerase transcription factors or target regulatory regions. Interestingly, the gene coding for PTPRD (protein tyrosine phosphatase receptor type delta), a STAT3 inhibitor, was found to be hypermethylated compared to non-mutant *TET2* NK-LGL or normal NK cells.

*TET2* mutations are common in both myeloid blood malignancies (acute myeloid leukemia/myelodysplastic syndrome, chronic myelomonocytic leukemia) and T-cell lymphoma, particularly in angioimmunoblastic T-cell lymphoma, which raises questions as to the original cell that underwent the *TET2* mutation in NK-LGL leukemia. In whole exome sequencing studies in 3 out of 6 patients analyzed, we showed that *TET2* was mutated not only in NK cells but also in myeloid precursors, suggesting a potential driver role of *TET2* mutation (29). This may explain cases of LGL leukemia association with a myelodysplastic syndrome or acute myeloid leukemia (39).

## Clinical Characteristics of NK-LGL Leukemia

T-LGL and NK-LGL leukemia share both pathophysiology, clinical and biological presentation (**Table 1**). The median age of LGL leukemia onset is 60 years with a sex ratio of 1:1. Its course is indolent with an overall 10-year survival rate of 70% (1). Massive hepatosplenic and bone marrow infiltration of NK-LGLs and rapidly progressive NK cell blood lymphocytosis, is related to aggressive NK cell leukemia, a rare and distinct entity with a poor prognosis (42). Symptoms are mainly due to infections (mouth ulcers, ENT or lung infections, severe sepsis) secondary to severe neutropenia which is the most common cytopenia. Neutropenia is less frequently observed in the NK subtype (29% in T LGL leukemia, as compared to 61% in NK LGL leukemia) (29, 40). Infectious complications are responsible for the majority of disease-related deaths (3-7%) (29, 40). Opportunistic infections are rare and secondary to immunosuppressive therapy. Twenty percent of patients are transfusion dependent. Thrombocytopenia is rare and moderate. LGL leukemia can be complicated by pure red cell aplasia or bone marrow aplasia. On clinical examination, splenomegaly is observed in 25% of cases (41), whereas hepatomegaly is slightly less frequent and peripheral adenopathies are rare.

**Table 1:** Comparison of clinical characteristics between T-LGL and NK-LGL leukemia.

	NK-LGL leukemia	T-LGL leukemia
	Poullot (n=70) [Ref: (40)]	Bareau (n=201) [Ref: (41)]
Lymphocytes > 4G/L	56%	51%
Median LGL (G/L)	2.1	1.71
LGL <1G/L	29%	55%
LGL > 7G/L	7%	4%
Neutrophils < 1.5G/L	29%	61%
Neutrophils < 0.5%	9%	26%
Anemia < 11g/dL	18%	24%
Anemia < 8g/dL	9%	6.6%
Thrombocytopenia <150G/L	20%	19%
Thrombocytopenia < 50G/L	4%	1%
Autoimmune diseases	24%	33%
Rheumatoid arthritis	7%	17%
Seronegative arthritis	14%	8%
Polymyositis	3%	0%
Autoimmune hemolytic anemia	6%	<7%
Idiopathic thrombocytopenic purpura	7%	<7%
Vasculitis	4%	3%
Solid cancers	13%	5%
Associated blood disorder	11%	8%
B-cell lymphoma	3%	–
Myelodysplastic syndrome	3%	–
Acute myeloid leukemia	3%	–
Myeloproliferative syndrome	4%	–

LGL leukemia may be associated with autoimmune diseases, such as connective tissue disorders or vasculitis. Rheumatoid arthritis is the most common condition seen in individuals with LGL leukemia, although slightly less frequent in NK-LGL leukemia (40, 41). These diseases can precede diagnosis of LGL leukemia. In autoimmune disease settings, reactive NK cell proliferations may also be observed. Moreover, some connective tissue disorders such as lupus and Gougerot-Sjögren syndrome can have overlapping clinical characteristics such as neutropenia, pure red cell aplasia and splenomegaly that can make the diagnosis of LGL leukemia difficult. Biological markers of autoimmunity such as polyclonal hypergammaglobulinemia and presence of positive rheumatoid factors are common and the signs of a chronic antigenic stimulation mechanism (40). Moreover, there have been reports of LGL leukemia concomitant with another hematological malignancies, either of myeloid or lymphoid origin. MGUS is more common than in the general population (16%) (40, 43). LDH and beta-2 microglobulin levels are high in 36 and 66% of cases respectively (40). Concomitant association with solid cancers has also been described (44).

## The Contribution of Flow Cytometry and Bone Marrow Biopsy to the Diagnosis of NK-LGL Leukemia

The vast majority of NK-LGL leukemia cases harbored a cytotoxic CD16^high^CD56^low^CD57+/- profile (29). Therefore, NK leukemic cells most often display a uniform CD16^high^ profile whereas normal NK cells are characterized by heterogeneous CD16 expression due to the coexistence of different NK subtypes. High CD16 expression is not sufficient to affirm NK clonality but provides an invaluable clue in the diagnostic procedure. CD56 is expressed by some activated T cells and in T-LGL leukemia and is therefore not a good marker of NK clonality. CD57 is positive in the majority of cases, associated with a memory profile (29, 31).

While normal NK cells display a CD2+/CD5-/CD7+ phenotype, clonal NK LGLs are frequently CD5dim/CD7dim. NK-LGL leukemic cells partially express CD8 with an intensity that is markedly lower than in T-LGL leukemia. However, CD8 cannot be used to distinguish NK-LGL leukemia from normal NK cells which exhibit low CD8 expression levels (3, 45). KIR phenotyping represents a major advance in NK-LGL leukemia diagnosis. However, this multiparameter analysis is complex and requires an expertise only available in some reference centers. NK-LGL leukemic cells show a restricted activated KIRs expression (15). Thus, inhibitory CD158a, CD158b and NKB1, expressed ubiquitously in normal NK cells, are very rarely expressed in NK-LGL leukemia (45). NK-LGL monoclonal proliferations express CD94 lectin with inhibitory NKG2A (15, 45), forming the CD94/NKG2A heterodimer, with a markedly higher MFI than that observed in normal or reactive NK cells. To a lesser extent, underexpression of CD161 (3) and natural cytotoxicity receptors (15), in particular NKp30 and NKp44, is more often found in NK-LGL leukemia than in NK-LGL polyclonal proliferations.

Bone marrow biopsy may contribute to ascertain the diagnosis in atypical presentations, specifically with a low LGL count (< 1G/L), an irrelevant phenotype, a marrow hypoplasia or pure cell aplasia. In paraffin sections, diffuse interstitial medullary infiltration by LGLs is found in more than 90% of cases, with a TiA1 and granzyme immunostaining. It is noteworthy that CD3 can sometimes be positive in immunofluorescence staining because of the presence of a CD3delta subunit on the NK cells, which binds to paraffin on immunolabeling. Moreover, LGLs are grouped into clusters of at least 8 TIA-1+ lymphocytes or at least 6 granzyme B+ lymphocytes. These LGL clusters may be associated with nodules of B cells surrounded by non-clonal CD4+ T cells. Intrasinusoidally, LGLs dysplay a linear TIA1+/granzyme B/+ network in close contact with antigen-presenting cells (46, 47)

## Contribution of Genomic analysis. Proposal for an NK-Cell Clonality Score

Identification of recurrent mutations in T- and NK-LGL leukemia provided strong arguments for NK clonality, and ultimately enabled true NK-LGL leukemia to be distinguished from reactive NK-LGL proliferations. Mutational screening is more accurate than KIR receptor repertoire analysis. The frequencies of the different mutations are shown in **Table 2**.

**Table 2:** Phenotypic and mutational profiles of NK-LGL leukemia in the French cohort and USA cohort.

	French cohort n=46 LGL and 68 Reactive NK [Ref: (29)]			USA cohort n=63 [Ref: (31)]
	Training set N=28 LGL	Validation set N=18 LGL	Reactive NK N=68	
NK count >1G/L	68%	83%	19%	NA
KIR restricted phenotype	86%	78%	6%	NA
CD94/NKG2Ahi	68%	61%	15%	NA
*STAT3*	26%	28%	0%	29%
*STAT5b*	8%	0%	0%	0%
*TNFAIP3*	9%	11%	0%	10%
*TET2*	35%	33%	8%	28%
*CCL2*	NA	NA	NA	22%

NA, Not Applicable.

The first major recurrent mutation initially identified in T-LGL leukemia was a *STAT3* function gain mutation found in 27-33% of NK-LGL leukemia cases (29, 31, 48). The STAT3 mutations are located in the SH2 domain within exon 20 and 21, Y640F and D661V accounting for two-thirds of mutations (17). The *STAT5B* mutation is less common, present in 5% of LGL leukemia cases (29, 49). The *TNFA1P3* mutation is particularly observed in cases of LGL leukemia associated with rheumatoid arthritis, and in 5-10% of NK-LGL leukemia cases (21, 31, 48).

In 2021, using high-throughput sequencing we and others have identified a *TET2* mutation in 28 to 34% of NK-LGL leukemia cases, constituting a new strong diagnostic marker (29, 31). *TET2* and *STAT3* mutations are generally exclusive and appear to be associated with two different NK phenotypic and functional profiles: the *STAT3* mutation is more often found in CD16high/CD57low, or cytotoxic memory NK-LGLs, while the *TET2* mutation is more commonly associated with the CD16low, or regulatory cytokine profile. The transcriptome expression profiles analyzed by C. Pastoret et al. in *STAT3*- and *TET2*-mutated patients are quite distinct, confirming the existence of two different subgroups. Moreover, a genotype/phenotype correlation was observed, reflecting the strong impact of these mutations in the pathophysiology of LGL leukemia; *STAT3*-mutated patients have a higher incidence of neutropenia (25, 37, 48) while *TET2* mutant patients have a higher incidence of thrombocytopenia (29, 31). *STAT5B* N642H mutated patients develop more aggressive disease (50).

**Figure 2:**
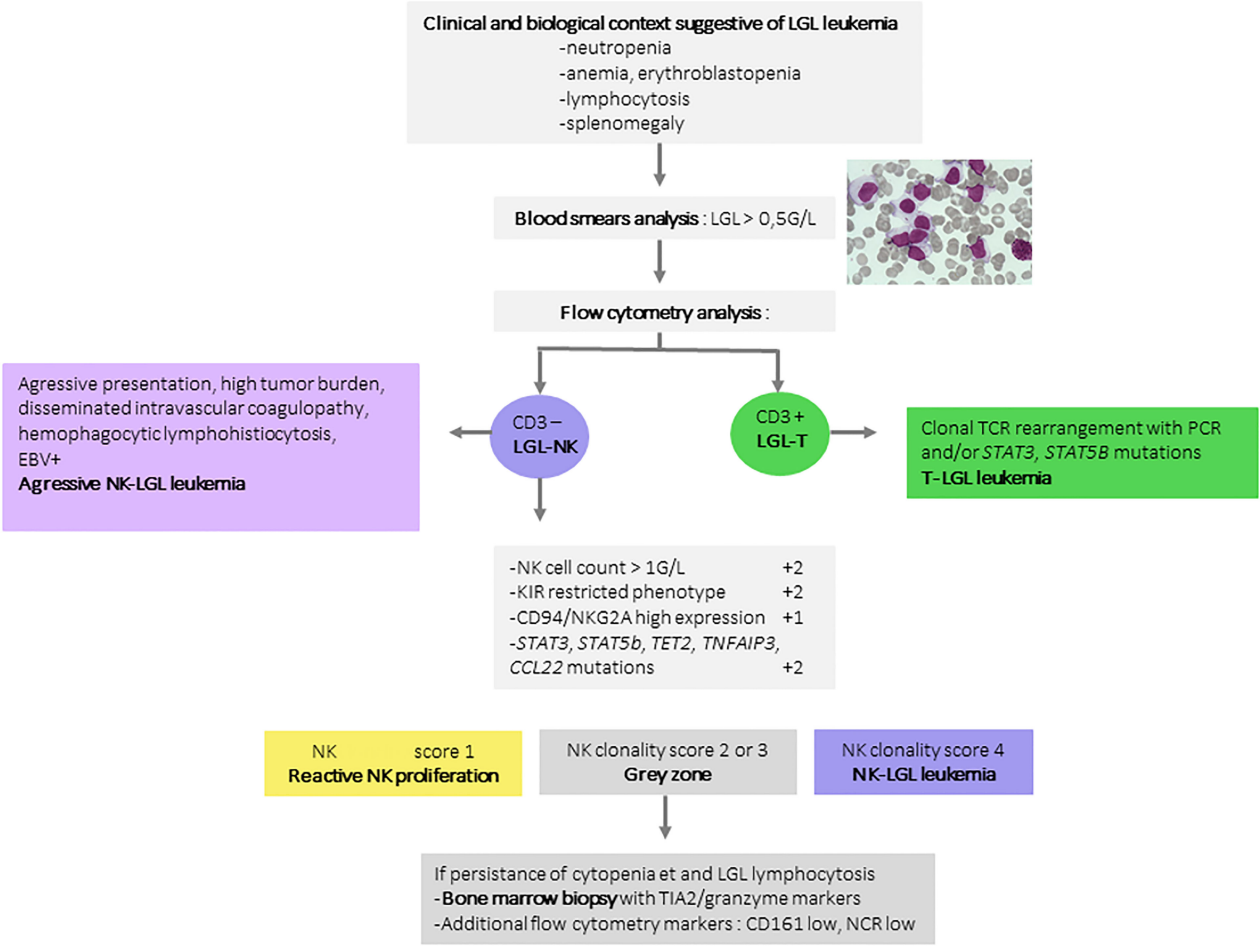
Diagnosis algorithm for NK-LGL leukemia. A large granular lymphocyte count greater than 0.5G/L is the first element mandatory for the diagnosis of NK-LGL leukemia. T and NK-cell LGL are distinguished based on the expression of CD3. The proof of clonality is often challenging in NK-cell LGL. In these conditions, the proposed diagnostic score assigns 2 points for a restrictive KIR phenotypic profile, 1 point for CD94/NKG2A hyperexpression, 2 points for *STAT3, STAT5b, TET2, TNFAIP3*, or *CCL22* mutations. These three elements represent the most compelling arguments for clonality. In case of a score higher than 4, the diagnosis of LGL-NK leukemia can be confirmed. A score between 2 and 3 should prompt discussion of the evaluation of other NK markers such as low CD161 or low NKp30 and 44. In this situation, a bone marrow biopsy is recommended. LGL, large granular lymphocyte leukemia; KIR, Killer-cell Immunoglobulin-like receptors; STAT3, Signal transducer and activator of transcription 3; TET2, Ten-eleven-translocation 2; TNFAIP3, Tumor Necrosing Factor Alpha Induced Protein 3; CCL22, C-C Motif Chemokine Ligand 22.

However, *TET2* mutation is not restricted to LGL leukemia and has been identified in angioimmunoblastic lymphoma and other T-cell lymphomas. Overall, in two-thirds of NK-LGL leukemia cases, a recurrent mutation contribute to the diagnosis. In routine practice, a high-throughput sequencing panel for T-cell lymphoma including screening for *STAT3, STAT5B, TNFAIP3, CCL22* and *TET2* mutations can thus be used for the diagnosis of NK-LGL proliferations. We proposed a prognostic score based on biological criteria ranging from 0 (low probability of clonality) to 7 (high probability of clonality) in settings suggestive of LGL leukemia (36). The criteria yielding two points each were as follows: i) NK cell count > 1G/L, ii) KIR receptor restriction defined by a low expression of at least two KIR receptors (CD158A < 9% of NK cells, CD158B < 12%, and/or NKB1 < 4%), and iii) presence of a somatic mutation of *STAT3, STAT5b, TET2* or *TNFAIP3*. A high expression of CD94 or NKG2A (>77%) carries an additional point. A score higher than or equal to 4 has a sensitivity of 83% and a specificity of 96% for NK-LGL diagnosis and a score of under 2 discounts the diagnosis with a negative predictive value of 95%. This score was validated on a cohort of 38 patients (18 LGL and 20 reactive conditions), yielding a positive predictive value of 100%. Only one LGL according conventional criteria was reclassified as reactive condition according the NK score **(** **Table 2** **).** Finally, mutations in the *CCL22* gene are also described in 20% of LGL-NK leukemias, specific to the NK subtype, and exclusive of other mutations (38). A diagnostic algorithm for LGL-NK leukemias is proposed in **Figure 2**.

## Therapeutic Approaches in NK-LGL Leukemia

The indolent course of LGL leukemia allows a watch and wait attitude at initial diagnosis in one third of patients. However, two thirds of patients will be eventually treated mainly due to neutropenia related infections or symptomatic anemia. The treatment indication can also be discussed in case of associated and symptomatic disease. It should be noted that there are no studies evaluating specific treatment of the NK-LGL leukemia subtype, as patients with NK-LGL leukemia were included with T-LGL patients with no distinction made. Immunosuppressive drugs such as methotrexate, cyclophosphamide and ciclosporin constitute the backbone of first-line treatments. Complete response rates at 4 months are low (around 16%). A prospective randomized study of first-line therapy (51), comparing methotrexate with cyclophosphamide, is currently underway. Relapse is frequent, occurring within a median time of 9 to 29 months (51, 52). Ciclosporin is more readily used in aplastic forms or pure red cell aplasia. Treatment must be maintained for at least one year in order to prevent early relapse.

In frequent cases of LGL leukemia that are refractory to immunosuppressive agents or in early relapse, alemtuzumab, an anti-CD52 antibody, alemtuzumab, which is also the treatment of choice for T-cell prolymphocytic leukemia, was tested in LGL leukemia with several response cases (53–55). A gamma chain inhibitor of the cytokine receptors IL2 and 15, BNZ-1 (56), was shown to induce *in vitro* a reduction in STAT3 and ERK phosphorylation in NK- and T-LGLs, and to induce apoptosis of T-LGLs. A phase I/II is underway (57). The use of therapies targeting the JAK/STAT pathway constitutively activated in LGLs appears promising. For example, remission was achieved with tofacitinib in a small number of refractory T LGL leukemia patients (53–55, 58), and likewise with ruxolitinib. No…..e remission rates induced with demethylating agents in cases of TET2 mutated angioimmunoblastic lymphoma (59) should prompt an assessment of their efficacy in LGL leukemia bearing the TET2 mutation.

## Conclusion

It is now possible to propose a more precise classification of NK-LGL leukemia and discard the term chronic NK lymphocytosis. Proof of clonality of NK-LGL leukemia is crucial given the frequency of reactive NK-LGL proliferations. The identification of a phenotypic restriction in KIRs combined with identification of a *STAT3, STAT5B, TET2, TNFAIP3*, and *CCL2* mutations constitute strong arguments to confirm NK clonality in most cases. Targeted JAK/STAT pathway therapies and demethylating agents in the case of *TET2* mutation represent promising therapies that warrant assessment in prospective studies in order to reduce the relapses frequently reported after immunosuppressive therapy.

## Author Contributions

GD, TL, and TM wrote the manuscript. All authors contributed to the article and approved the submitted version.

## Funding

TM is supported by the “Association pour le Développement de l’Hématologie Oncologie” (ADHO).

## Conflict of Interest

The authors declare that the research was conducted in the absence of any commercial or financial relationships that could be construed as a potential conflict of interest.

## Publisher’s Note

All claims expressed in this article are solely those of the authors and do not necessarily represent those of their affiliated organizations, or those of the publisher, the editors and the reviewers. Any product that may be evaluated in this article, or claim that may be made by its manufacturer, is not guaranteed or endorsed by the publisher.
